# “I can’t stand it…but I do it sometimes” parental smoking around children: practices, beliefs, and conflicts – a qualitative study

**DOI:** 10.1186/s12889-020-08863-7

**Published:** 2020-05-14

**Authors:** Vicki Myers, Eimi Lev, Nurit Guttman, Efrat Tillinger, Laura Rosen

**Affiliations:** 1 Department of Health Promotion, School of Public Health, Faculty of Medicine, Tel Aviv University, Ramat Aviv, Tel Aviv, Israel; 2 Department of Communications, Faculty of Social Sciences, Tel Aviv University, Tel Aviv, Israel; 3 Department of Communications, Gordon College of Education, Haifa, Israel

**Keywords:** Secondhand smoke, Tobacco smoke exposure, Children, Qualitative, Parental behavior

## Abstract

**Background:**

Many parents continue to smoke around their children despite the widely known risks of children’s exposure to tobacco smoke. We sought to learn about parental smoking behavior around children from parents’ perspective.

**Methods:**

Semi-structured interviews were conducted with 65 smoking parents or partners of smoking parents of children up to age 7, to learn about home smoking rules, behaviours performed to try to protect children, and smoking-related conflicts, from parents’ perspective. Interviews were recorded and transcribed and thematic analysis performed. Recruitment was challenging due to the sensitive nature of the topic.

**Results:**

Many parents described smoking around their children in certain areas of the home, outdoors, and in what they consider to be open or ventilated areas. Participants emphasized efforts to protect their children and described various mitigating practices but held mixed views as to their effectiveness. Parents had different conceptions of which areas or distances were considered ‘safe’. Many smoking parents described conflicts both internal and with other family members regarding the protection of children. Some parents who continue to smoke around their children despite understanding the health risks felt powerless to effect change, as well as being uncertain as to the effectiveness of their protective strategies; others were aware but reluctant to change.

**Conclusion:**

Findings shed light on some of the difficulties faced by smoking parents and obstacles to maintaining a smoke-free environment for their children, providing insight for the type of information and support required to help parents better protect their children from exposure to tobacco smoke. Awareness of health risks associated with secondhand smoke was demonstrated, yet parents in smoking families were confused regarding which rules and behaviours best protect children from exposure to tobacco smoke. Parents were sometimes aware that their smoking ‘rules’ and mitigating practices were limited in their effectiveness. Guidelines should be provided explaining how and when exposure occurs and how to keep children safe.

## Introduction

While some progress has been made in the field of smoke-free homes, many parents continue to smoke in the home or in their children’s environment despite the widely known risks of children’s exposure to tobacco smoke [1–3]. The number of children regularly exposed to cigarette smoke is estimated to be around 40% worldwide [4]. Many women stop smoking in pregnancy, but resume smoking several months after giving birth [5]. Exposure to tobacco smoke is causally related to chronic ear infections, lower respiratory infections, exacerbation of asthma and sudden infant death syndrome [6], as well as lifelong cardiovascular outcomes [7].

In Israel, the smoking rate has remained about 20–22% in recent years [8]. While restrictions have been brought into law regarding smoking in public places, including schools, train platforms, bus stops, restaurants, swimming pools, these are not always enforced, and no legislation exists to protect children from tobacco smoke in the home or car. Indeed a 2017 report from the Israeli Ministry of Health found that between 25 and 35% of teenagers reported being exposed to tobacco smoke at home, and a biomarker study examining urine cotinine in children aged 5–14 in 2015–16 found substantial levels of detectable cotinine (a metabolite of nicotine) [9].

Attempts have been made to determine what motivates parents to continue exposing their children to what is widely known to be a dangerous substance, and to illuminate the obstacles to eliminating tobacco smoke exposure. A review of qualitative studies described barriers including lack of knowledge, poor awareness of risks, social norms, addiction, and the practicalities of caring for small children; while motivators to having a smoke-free home may include awareness of risk, guilt and avoidance of stigma, wanting to be a responsible parent and wanting to protect others’ health [10].

Although parents typically would like to protect their children from tobacco smoke, many continue to smoke around their young children. This study aimed to better understand parental smoking behaviour around small children, using qualitative methods. While previous qualitative research has examined smoking parents’ beliefs and attitudes to smoking around children [11], in this study we additionally examined how parents feel about protective actions they take and whether they think that their actions are indeed effective in protecting their children.

Study questions included: 1) in what circumstances do Israeli parents smoke around children in and around the home environment; 2) in what ways do parents try to protect children from tobacco smoke; 3) how do smoking parents feel about their smoking behaviour and mitigating practices; and 4) what are parents’ perceptions of self-efficacy regarding protecting children from exposure to tobacco smoke.

## Materials and methods

Participants were recruited from Meuhedet Health Care Services, by word-of-month, and via the snowball technique. Purposive sampling was used to select clinics in different geographical areas in central Israel, to ensure the recruitment of participants from a variety of socio-economic (low, medium, and high), ethnic (Israeli or non-Israeli born) and religious (secular, religious, and ultra-religious) groups within the Jewish population. Inclusion criteria were parents in families in which at least one parent smoked, with a child up to age 7 years. We originally aimed to recruit parents of children up to age 3 but expanded the inclusion criteria to boost recruitment. Children up to age 7 are of interest as they spend more time at home and around their parents than older children. Ethical approval was obtained from the Tel Aviv University Ethics Committee, the Laniado Hospital Helsinki Committee (0014–11-LND), and the Helsinki Committee of the Israel Ministry of Health (MOH: 920090057). Participants provided signed informed consent and received a gift voucher worth approximately $30 to compensate them for their time.

Face-to-face interviews were conducted in Hebrew between September 2011 and August 2012, mostly in participants’ homes, and interviewers were trained by a medical sociologist (ET). Semi-structured interview guides comprising approximately 50 questions, mostly open-ended, were developed based on previous works (Personal communication, Elizabeth Gonzales, Project KISS 2009; Personal communication, Robyn Keske, 2012, Project: Breathe Free for Kids; Personal communication, Deborah Ritchie, 2012, Project: REFRESH). Interviews were audio-recorded and transcribed verbatim.

This was part of a wider study which investigated smoking in the context of parenting [3, 12, 13]. The interview guide ran as follows: Parents were first asked to describe their family situation, how smoking fits into their life, where they usually smoke (describing the exact place in or outside the home), who else in the family smokes, whether there was anything they don’t like about smoking, and to describe home smoking behaviour. The current study relates to parents’ descriptions of smoking rules (“Do you have any rules regarding smoking around the child(ren)? If so what are they?”), which specific behaviours they perform to protect the child from exposure and to describe any conflicts within the family related to smoking. Specific questions included “Do you sometimes smoke in the presence of the child? If so in what circumstances?” Parents were also asked what they know about secondhand smoke, and how they think it affects children and how they feel about protective actions they take.

Data were transcribed in Hebrew and accuracy of transcripts was checked. Participant names were changed to numeric codes to maintain anonymity. Thematic analysis was employed as an iterative comparison process within and across participants [14, 15]. This type of analysis enables analyzing and reporting themes within the data in a detailed manner. The analysis involved the following steps. First parts of the text which related to the present analysis were identified. Next each text segment was assigned a descriptive code, and codes depicting related topics were grouped and classified into relevant themes and categories. Since participants were asked to describe home smoking rules and practices, these questions provided an overall framework for the analysis or ‘deductive’ and ‘descriptive’ broad categories [16]. However, the analysis aimed to identify categories and organizing themes that emerged as prominent or divergent as they relate to each of these topics such as particular situations or places, for which different ‘rules’ were applied, as well new issues that emerged such as conflicts with others, normative conceptions, or beliefs in the efficacy of the protective measures and self-efficacy [17]. A summary report including illustrative quotes was written in Hebrew and translated into English by a professional translator. We then went back to the Hebrew transcripts to confirm themes and identify excerpts which reflected the experiences and views of the participants in relation to the different topics. This stage was conducted independently by two researchers (VM, EL) for inter-observer reliability. The analysis was subsequently reviewed and refined by all members of the research team to reach consensus on interpretation of themes.

### Participants

Sixty-five parents from 65 families took part in the study, out of 123 who originally expressed interest (53%). Reasons for non-participation were being unavailable for interview or unreachable (*n* = 39), having quit smoking (*n* = 2), did not want to participate after receiving more details (*n* = 10), did not wish to be recorded (*n* = 1), did not meet inclusion criteria (*n* = 1), or unknown reasons (*n* = 5).

The study sample comprised 48 mothers and 17 fathers, of which 54 smokers and 11 non-smoking partners of smokers. Mean age was 33.3 ± 4.8 years. The mean number of children per family was 1.91 (range 1–4). Around a third of smokers (*n* = 21) were light smokers (0–5 cigarettes/day); a third (*n* = 21) were moderate smokers (6–10 cigs/day); and a third (*n* = 22) were heavy smokers (more than 10 cigs/day).

## Results

### Analytic results

The analysis yielded the identification of four major issues related to parental smoking practices around children:

1. Rules concerning in which places and situations parents smoke around their children;
2. Behaviours parents perform in an effort to protect their children from exposure to tobacco smoke, and how they feel about the efficacy of these mitigating practices;
3. Conflicts and negative emotions experienced by smoking parents, within themselves and with others with whom they interact regarding smoking around children; and.
4. Self-efficacy concerning curbing parental smoking behaviour around children.

#### Rules concerning in which places and situations parents smoke around their children

Many parents described home smoking rules self-enforced in an attempt to protect children. Smoking rules ranged from the more restrictive, such as never smoking inside or around the home at all, followed by the less stringent smoking on the outside balcony with the door to the house closed. Less restrictive rules included smoking on the balcony without closing the door; smoking only in a designated room or on an internal balcony; smoking at the window; or smoking in the home when the children are not present.

#### On the balcony

Many participants reported smoking on the porch or balcony, which is usually connected to the main living room; however some parents who reported smoking on a balcony revealed on further questioning that they actually smoke in some enclosed part of the home such as the utility balcony, which is an enclosed room with a window where the laundry is hung. Smoking on an indoor utility ‘balcony’ was often not considered to constitute smoking inside the home, as described here: “At home we have a utility balcony, it’s usually there, there’s no way I’ll smoke in the house when the children are there.”

#### In the car

A range of more and less restrictive rules was also seen regarding smoking in the car. While many parents reported smoking in the car, a common rule was refraining from smoking in the car when children are present; or stopping smoking some time before the children get in the car (this time was sometimes defined as, for example, 10 min, an hour, and was sometimes undefined); less common and more restrictive was no smoking in the car at all. Others described strong feelings against smoking in the car at all, basing their objection on the smell which remains in the car.

#### Outdoors

Rules about outdoor smoking were less common – smoking outdoors in the vicinity of children was considered safe - or at least safer than smoking indoors – by most parents. Most parents talked about smoking around their children in outdoor spaces including in the garden, the street, shopping centers, parks, playgrounds, the beach and others. Rules about smoking around children outside often involved an element of distance. Smoking while walking with a child in the stroller was commonly described, although some felt this was an unacceptable practice.

#### Behaviours parents perform in an effort to protect their children from tobacco smoke exposure (Table 1)

Most parents knew that secondhand smoke is dangerous and involves health risks for children and made attempts to protect their children. Yet many parents did not know about third-hand exposure, that children can still be exposed to smoke after the cigarette has been extinguished.

**Table 1:** Protective behaviours

Theme	Quotes
Rules about smoking at home (and definition of ‘in the home’)	“At home we have a utility balcony, it’s usually there, there’s no way I’ll smoke in the house when the children are there.”
	“I smoke only on the balcony and I always close it off (from the rest of the house)”
Limitations of when/where smoking is acceptable: Car	Interviewer: Do you ever smoke with the kids in the car? Participant: No, that’s the limit.”
	“Smoking a cigarette in the car while the smoke and the cigarette odor remains, it seems shocking to me.”
Limitations of when/where smoking is acceptable: Stroller	“I regularly smoke while strolling with the carriage because cigarettes are already part of my bag of ‘supplies’.”
	“A lot of mothers stroll with the baby carriage and smoke freely. No way will I do that”
Maintaining distance	“I smoke next to them outside, but I don’t smoke ‘on top of their heads’.”
Protective behaviours: smoke-free home	“I don’t smoke inside the house; even if I smoke outside the house I make sure the door is closed so that no smoke comes in.”;
Protective behaviours: at the window	“I smoke at the window…my whole head is outside, I’m almost falling out”.
Protective behaviours: personal hygiene	“I change my shirt after smoking, thoroughly wash my hands, rinse my mouth with mouthwash and try very hard to have no smoke odor on me.”
Greater importance of protecting smaller children	“So while he’s small it’s very important for me that he not be near an environment of smokers… suddenly he seems like a big boy, so it seemed like it was OK to smoke near him”
	“When his oldest daughter was a baby, he’d protect her from friends and tell them to keep their distance (when smoking), or remove her from the scene.”
Confidence in protective measures	Participant: “First of all I smoke obviously with all the windows open and if I need to pick up the kids, then I won’t smoke in the car an hour before… I always open the windows, but I don’t go crazy about it, …” Interviewer: “Do you think it’s effective to reduce exposure to passive smoking?” Participant: “Opening the windows? …Of course it is!”
Uncertainty regarding protective measures	“I don’t really think that any of it reaches her when we smoke and walk with the stroller, it doesn’t seem reasonable that it would reach her, but it could be that I don’t know enough”.
	Participant: “If I’m on the way from work to pick up the children then I’ll smoke my cigarette at the start of the journey and then the window will be open until I get there Interviewer: And do you think that’s effective? Participant: No, yes and no. It doesn’t completely get rid of it, it might reduce it.”
Acceptance of partially effective protective measures	“It’s better than nothing. Obviously I know that the odor sticks to things to a certain extent. For sure it still has a certain effectiveness, airing out…”
	“If I smoke in the car on my way to picking up the kids, I say to myself: ‘OK, it’ll air out by the time I put them in the car’. But that’s a bunch of bull. It doesn’t totally disappear, even if you leave the window open.”
	“I also do it, but it’s bogus. It absorbs into the upholstery. I do it only to ease my conscience.”
	“When I travel with ‘A’ in the carriage I open the overhead protective covering so that the smoke goes over it and not beneath it. So he’s somewhat exposed; sometimes he even coughs a bit.”

Different measures were described both in an attempt to protect children from one’s own smoke; and from the smoke of others. Parents reported adopting various strategies in order to reduce their children’s exposure to tobacco smoke, ranging from attempting to completely prevent the passage of smoke (for example by closing the door or window between child and smoker) or even preventing the children from witnessing the act of smoking; to harm reduction such as opening windows for ventilation while smoking. The main practices adopted by parents were: Creating smoke-free spaces, separating smokers and smoke from children (by time and space), using physical barriers such as a door or a stroller cover, and personal hygiene practices such as brushing teeth and changing clothes. In the home, parents sometimes moved in order to smoke: they went outside, to a balcony, to a different room, or stood near a window. Parents emphasized the effort they make to protect their children from exposure to tobacco smoke by smoking in their designated smoking place, closing the door “I don’t smoke inside the house; even if I smoke outside the house I make sure the door is closed so that no smoke comes in.”; or sticking their head out the window.

Parents who smoked on their balconies had a variety of approaches for dealing with children while smoking there: some welcomed their children to join them while they were smoking, others tried to prevent their children from joining them, while some went in and out of the house itself while smoking.

Outdoors, parents tried to protect children by distancing, either moving away from the child, walking in front of or behind the stroller or by redirecting the smoke so that it didn’t reach the child.

A greater emphasis was found placed on protecting babies and younger children, who are often considered more vulnerable and in need of greater protection compared to older children, for example one mother expressed this difference: “So while he’s small it’s very important for me that he not be near an environment of smokers… suddenly he seems like a big boy, so it seemed like it was OK to smoke near him”.

Additional mitigating strategies included personal hygiene used in an attempt to reduce children’s exposure to smoke, including washing hands, changing clothes and brushing teeth after smoking and before interacting with the children. This emphasis on trying hard to protect the children was a recurring theme.

#### Beliefs and perceptions about the efficacy of parental practices used to protect children from tobacco smoke exposure

When discussing the different things they do to protect their children from tobacco smoke, parents expressed different attitudes – some were convinced their actions successfully protect their children, some were unsure, while others considered them to be of limited effect.

Those that expressed certainty felt confident that their efforts to protect their children were successful, for example opening all the windows in the car, while smoking or smoking out of the window at home.

Some were unsure how effective their actions were, for example when smoking while walking with the stroller, or unsure about the time it takes to air out the car, after smoking and before collecting the children.

Others considered their mitigating actions to be somewhat effective and reduce harm but not wholly effective in preventing exposure. For example one mother mentioned raising the hood of the baby carriage while smoking to prevent smoke reaching him, and yet the baby coughing sometimes: “So he’s somewhat exposed”. Another example was airing out the car after smoking, which was viewed by some parents as not entirely effective since the smoke absorbs into the upholstery or the smell remains in the car. Thus, these parents were aware that their ‘rules’ or mitigating practices might reduce the harm, but nonetheless their children were not fully protected.

Some parents explicitly said that they continued to perform mitigating practices even when not convinced that they are effective, for example airing out the car after smoking, saying “it’s better than nothing”.

It appears that many parents continue to smoke next to their children, despite being aware of the existence of exposure, evoking a sense of resignation among the interviewees.

#### Negative emotions and conflicts with self and family members (Table 2)

Parents described conflicts that arise, both internally, between their desire to be good parents and their behaviour that causes their children to be exposed to tobacco smoke; and conflicts with family members over the need to protect the children. In later stages of the interview some interviewees revealed complex positions and feelings while permitting an open discussion on this sensitive topic –sometimes themselves presenting the gap between striving to be a good parent/wanting to keep their children healthy and the need to smoke in terms of a conflict.

**Table 2:** Conflicts

Theme	Quotes
Self-criticism/ Being a good vs bad parent	“I can’t stand it [smoking while walking with the stroller], but I do it sometimes. It’s out of fatigue, those moments of fatigue. I always look at myself with a critical eye; on the other hand I also do it about twice a week.”
	“It makes me feel bad and I know it’s bad. I get so mad at myself but…it’s a conflict, a huge conflict… I mean it goes against everything that… as a parent you want only good for your children, and here you’re sticking poison in their face….”
	“I think it means being a bad father…. It doesn’t make them bad people just because they smoke. What I meant was the bad aspect of smoking…but I will never smoke next to my children, even when they’ll be 10 years old.”
Acceptance of imperfection – no guilt	“I’m not sorry for smoking nor am I trying to obtain anyone’s approval. I don’t have guilt feelings over smoking. That doesn’t mean that I need to smoke more. I’m aware that I need to do something”
Judgement of ‘others’	“I see it when they’re [others] looking at me. When I’m walking around with the carriage and I’m holding a cigarette… No, it doesn’t affect me…Maybe bothers me for a moment, but it passes.”
Conflicts with family	“There are arguments about that for example, about my mother, we argue about her smoking, me and my partner, it upsets her [my partner] that she [my mother] doesn’t make an effort not to smoke around the kids”
	Participant: “I fight with them [my parents] about it all the time…that they shouldn’t smoke next to the children.” Interviewer: “And what do they say?” Participant: “In my house I’ll do what I want.”… It happened once or twice, that I was there with the children and my dad lit up a cigarette, so I just took them and left.”

The gap between the aspiration for good parenting on the one hand and smoking habits that are actually harmful was found to generate a sense of conflict, or internal dissonance. The concept of being a ‘good’ or ‘bad’ parent arose in parents’ own terminology in the context of smoking around children, for example when discussing smoking in cars with children, one mother said: “It goes back to being a bad mother.”

A less dominant theme was that some parents expressed lack of guilt feelings regarding smoking around their children, and did not voice the existence of any emotional conflict, framing this in a realistic view of the world and its limitations. However parents who reported an ostensible lack of conflict and feeling fine with their habitual patterns of smoking behaviour were definitely aware of the social illegitimacy, for example one mother reported the looks of other people, a feeling of being watched by strangers.

#### Conflicts between family members regarding smoking practices (Table 2)

The smoking of other family members, such as grandparents, who smoke near the children was also raised as a source of conflict. This puts the parent in a difficult situation, having to decide whether to prioritise their parents’ wishes or their own wishes to protect the child. One parent defined the conflict as arising from the perceived lack of effort on the part of the grandparent to create a smoke-free environment for her grandchildren. “There are arguments about that for example, about my mother, we argue about her smoking, me and my partner, it upsets her [my partner] that she [my mother] doesn’t make an effort not to smoke around the kids.”

#### Perceptions of control of the child’s environment and self-efficacy to protect them (Table 3)

##### Perceived lack of control/low self-efficacy – ‘I would like to protect them but I can’t’

Some parents felt powerless or resigned to occasionally smoke in the presence of their children, for various reasons including not being able to leave small children alone, fatigue, practical considerations and logistics. A mother of a baby expressed it thus: “I can’t leave him alone for a minute, you understand?”, explaining why she smokes when walking with the baby in the stroller. These parents express the all-consuming nature of parenting small children and how it affects their smoking choices, and increases the likelihood of their smoking in the home, or around the children.

**Table 3:** Perceptions of control of the child’s environment and self-efficacy to protect them

Theme	Quotes
Perceived lack of control/low self-efficacy – ‘I would like to protect them but I can’t’	“I have this fantasy of not smoking next to them, but I don’t have that privilege. It’s like…smoking in secret. Or there might be an instance where I can do it without them being on top of me or next to me. So if I’m with them for 12 h a day on weekends it’s like hiding from them.”
	“(When I’m with) my baby I smoke only if he’s in the carriage. I can’t leave him alone for a minute, you understand? He′s still small.”
Perceived lack of control/low self-efficacy –practical barriers	“I try to go out on the balcony but it’s cold, and it sucks to stand out in the cold with a cigarette, so I smoke near them - it’s not great but it is what it is.”
Trying – making an effort	“I try very hard to have no smoke odor on me. I do everything to avoid anything reaching my daughter.”
	“I try not to smoke next to them, but they’re always coming in and out, in and out. I always tell them to go in and stay inside.”
Feeling in control – high self-efficacy	“You simply need to change the habit…From smoking in the car to not smoking in the car. It’s a habit that you have to give up. There are habits you need to get rid of – to decide and to give them up.”
	“We never smoke in the house, or in the car. Since our children were born, no such option exists.”

One parent talked of practical barriers to protecting the children such as the weather, which makes it difficult to smoke outdoors. The expression: “I try…but…” recurred in several interviews, expressing the conflict inherent for smoking parents. Many parents referred to their effort to ‘try’ to protect the children from tobacco smoke. Some felt it important to emphasize that they do ‘make an effort’ even if they do not always succeed.

##### Feeling in control – high self-efficacy

In contrast to parents who felt helpless about adopting practices that fully protect their children, some parents believed in their ability to protect their children in certain situations, to one extent or another, from tobacco smoke and exhibited a more internal sense of control. They offered their advice on how this can be done: an absolute ban on practices that might cause children to be exposed to tobacco smoke.

## Discussion

While most efforts in Israel’s tobacco control landscape have focused in recent years on taxation of tobacco products, smoke-free laws in public places (including schools), and most recently plain packaging and withdrawal of point-of-sale displays [18], exposure of children to tobacco smoke in the home sphere is not subject to regulation. The current study used interviews with smoking parents to address this sensitive issue, to improve understanding of parental smoking around the home.

In the current study we describe circumstances in which parents smoke or refrain from smoking around their children in a sample of Israeli parents, in particular their home smoking practices, strategies they employ in an attempt to protect their children from cigarette smoke and how they feel about the efficacy of these mitigating practices. Though parents demonstrated awareness of health risks from tobacco smoke, confusion was seen regarding actual exposure and risk in different circumstances. Furthermore, while some parents mentioned lingering odor in the car, or smoke absorbing into upholstery, there was not widespread awareness of the harms of thirdhand smoke when smoking in and around the home and car. Awareness of the harms of thirdhand smoke have been associated with greater likelihood of having a smoke-free home [19]. Parents reported on the rules and practices they apply in their smoking behaviour around children, which sometimes create a false sense of protection. We found different types of parental practices and perceptions with varying levels of confidence in the efficacy of their protective behaviour: those who believe they manage to mitigate effectively; those who try but believe their efforts are limited; and those who would like to mitigate but feel they cannot and consequently feel guilty.

The analysis points to several common behaviours that parents believe do not expose their children to cigarette smoke, when in fact exposure may still be occurring. Parents reported smoking around their children in various circumstances. In particular, smoking in the close vicinity of children outdoors, smoking on the balcony when the door to the house is open, smoking in the car when children are not present, and smoking by the window indoors or on an indoor ‘balcony’, all activities that could lead children to be exposed to tobacco smoke. Research has shown that smoking in another room or by a window is not sufficient to prevent exposure from occurring [20] and that only strict home smoking bans may be sufficient to reduce children’s exposure including third-hand exposure [21]. Additionally children of parents who sometimes smoke indoors and sometimes outdoors were shown to have much higher urine cotinine levels than those who always smoke outdoors [22], although even those who always smoke outdoors had twice as much cotinine as non-smoking controls. Indeed smoke levels can be high outdoors at close proximity [23]. Another study using biomarkers found that children of parents who try to protect their children from exposure to tobacco smoke had 5–7 times higher exposure than non-smoking households, though less exposure than those who smoked indoors and did not take protective measures [24]. Smokers have been shown to display an ‘optimistic bias’ and to overestimate the effectiveness of preventive behaviours [25, 26]. A recent study also found that smokers may rely on their sense of smell to assess exposure, giving them an inaccurate representation [3]. These findings together with our reports of parental smoking behaviour around children suggest that parents need to be made aware of exposure occurring both indoors and outdoors and in situations which they may not consider to involve exposure. Furthermore, parents were often unsure or unconvinced of the efficacy of their mitigating strategies, perhaps suggesting a lack of knowledge and a need for specific relevant information for this target audience of smoking parents.

The parents in the study also described interpersonal relationships and conflicts within those relationships which relate to smoking around children. Conflict was evident whether internally between the need to be a good parent and the need or wish to smoke; or with family members regarding their smoking habits around children. In a quantitative study of smoking parents, 29% reported experiencing role conflict, expressed as “being a smoker gets in the way of being a parent” [27]. This conflict may sometimes result in cognitive dissonance or a defensive need to believe that one’s efforts at protecting one’s child are sufficient. Alternatively role conflict may sometimes spur behavior change and readiness to quit as found in [27]. Parents in the current study exhibited different levels of self-efficacy and belief in their ability to make a change to the child’s exposure or their own behaviour. “Trying”, which seemed to generate a sense of capability allowing parents to reduce the dissonance between good parenting and damaging behaviour, appeared to perpetuate existing patterns of behaviour to a minimal extent of discomfort.

There was often a need to justify beliefs – many parents held strong views about what is and what is not acceptable concerning smoking around children, and spoke in strong terms of ‘other’ parents who do what they consider to be unacceptable, whether this was smoking in the car or with the stroller, or of being judged by others. For some parents these perceived social norms influenced their smoking behaviour, while others purported to disregard them. The tobacco control policies of a country may also influence parental smoking behaviour and smoking in the vicinity of children [28]. Whereas tobacco control laws are in place in Israel, and have been updated to include educational establishments [8], there are no laws regarding smoking around children in the home or car, and smoking continues to occur in many public spaces since enforcement is weak.

Several of the concepts revealed in our study reflect those reported in a review of qualitative studies which synthesized findings regarding barriers, motivators and enablers to smoke-free homes, for example lack of knowledge of effective strategies, guilt and stigma of being a smoking parent, the influence of others, issues of control [10]. A qualitative study of English mothers described a resistant dialogue, with smoking parents tending to attribute ill health to factors other than smoking [11]. While some parents in the current study did minimize the impact of smoke compared to other harms such as air pollution, knowledge of the health risks was evident. More widespread was the expression of making real attempts to protect children, and uncertainty regarding efficacy of protective measures. Parents – both in the current study and in previous research – make greater efforts to protect smaller babies and children, considering older children to be less vulnerable to tobacco smoke [5]. Another qualitative study of parents’ accounts of trying to protect their children from tobacco smoke talked about the constraints posed by living circumstances, which sometimes lead parents to feel powerless to make a change, and the complexity of social relationships and how these affect parental smoking behaviour [29]. The authors state that “all mothers reported taking steps to protect their children from SHS and smoking”(p.497) – similarly in the current study all parents mentioned things they do in an attempt to protect their children, whether it be smoking on the balcony and closing the door, asking their own parents not to smoke around the children, keeping their distance when smoking outdoors or trying to minimize the children’s exposure. The difference in the strategies employed may be affected by parents’ awareness or perceptions of exposure [3].

While previous studies have reported on what parents do to try to protect their children from exposure, our study went one step further asking how they feel about these behaviours, revealing that many parents feel a lack of confidence in the measures they take to reduce or prevent exposure. This lack of confidence may stem from different issues such as uncertainty as to the nature of exposure [3], but also from the difficulty involved in changing smoking habits. This could be addressed by providing clear guidelines to parents regarding the circumstances in which exposure occurs and how to best protect children. For example campaigns in the UK have used the ‘take 7 steps out(side)’ or ‘take it right outside’ slogan to make it clear that in order to protect children homes must be completely smokefree [30]. The US Environmental Protection Agency produced materials for community education advocating for smoke-free homes and cars [31]. No such campaign has been run in Israel. Furthermore messages about the dangers of exposure need to be reinforced to parents periodically as the tendency to protective behaviours seems to wane as children get older [5].

Some limitations should be taken into consideration: this study was performed in Israel with Israeli parents and is relevant to the context of Israeli society including cultural and social norms, as well as architectural differences relating to how people live (for example most people in Israel live in apartments in multi-unit buildings; some have a small outdoor porch/balcony; many have no outside space). This type of housing makes it difficult to completely eliminate smoking from the home environment when there are small children; furthermore living in multi-unit buildings means residents are exposed by or expose their neighbours when smoking on the balcony. We tried to obtain a mix of religious and non-religious participants and families from different geographical and socio-economic areas. As we see many of the themes reflect those found in similar studies conducted with smoking parents in other countries, we believe that there is common ground among smoking parents in different countries and cultures, and the main themes found here are not limited only to the specific cultural group interviewed here.

Results of this study shed some light on the circumstances surrounding children’s exposure to tobacco smoke in and around the home, in a sample of smoking parents. While some parents consider themselves as not smoking in the home, or around the child, they may in reality and on closer inspection smoke sometimes inside the home and in the child’s environment, unwittingly exposing them to tobacco smoke. Most previous qualitative studies of parental smoking behaviour were conducted in UK, North America, or Australia (see a comprehensive review by [10]), and the findings highlighted here are of course relevant to Israeli society, taking into account climate, cultural norms and architectural differences. However many of the views expressed by parents, for example feelings of guilt, conflicts with others, making an effort to protect children, were reflected in other qualitative studies from around the world, and may be representative of other smoking parents.

## Conclusions

The findings of this study offer an insight into parental smoking practices, perceptions and beliefs held by parents about children’s exposure and illustrate how parents are sometimes aware that their ‘rules’ and mitigating practices are limited. Parents described smoking around their children in certain circumstances, including in certain areas of the home, outdoors, and in what they consider to be open or ventilated areas. Mitigating practices were common and parents held mixed views as to how effective these practices are in protecting their children from exposure to tobacco smoke. Parents who continue to smoke around their children despite understanding the health risks may feel powerless to effect change, as well as being uncertain as to the effectiveness of their protective strategies. Incomplete knowledge about exposure and low self-efficacy may play a role. Better understanding of how and why parents smoke around their children can facilitate the design of interventions and creation of educational materials for parents to help them reduce children’s exposure to tobacco smoke.

### Implications for practice

There is no safe level of exposure to tobacco smoke. Lack of knowledge, misconceptions and confusion exist among smoking parents as to which rules and behaviours can best protect children from exposure to tobacco smoke. This can give parents a false sense of security that they are protecting their children when in fact exposure may still be occurring. Guidelines should be provided explaining how and when exposure occurs and how to keep children safe, emphasizing the importance of smoke-free homes and cars. Providers including pediatricians and well-baby clinics could be well placed to provide relevant information to parents. Armed with more comprehensive knowledge, smoking parents who are unwilling or unable to quit may feel more confident in their abilities to protect their children.

## Data Availability

The datasets analysed (transcripts) during the current study are not publicly available since they include personal details but could be made available in anonymized form from the corresponding author on reasonable request.

## References

[1] Orton S, Coleman T, Jones LL, Cooper S, Lewis S (2015). Smoking in the home after childbirth: prevalence and determinants in an English cohort. BMJ Open.

[2] Kinga BA, Patela R, Babba SD, Hartmanb AM, Freemanc A (2016). National and state prevalence of smoke-free rules in homes with and without children and smokers: two decades of progress. Prev Med.

[3] Rosen LJ, Lev E, Guttman N, Tillinger E, Rosenblat S, Zucker DM (2018). Parental perceptions and misconceptions of child tobacco smoke exposure. Nicotine Tob Res.

[4] Oberg M, Jaakkola MS, Woodward A, Peruga A, Pruss-Ustun A (2011). Worldwide burden of disease from exposure to second-hand smoke: a retrospective analysis of data from 192 countries. Lancet..

[5] Robinson J, Kirkcaldy AJ (2009). 'Imagine all that smoke in their lungs’ Parents’ perceptions of young children's tolerance of tobacco smoke. Health Educ Res.

[6] Department of Health & Human Services (2006). The Health Consequences of Involuntary Exposure to Tobacco Smoke: A Report of the Surgeon General.

[7] Raghuveer G, White D, Hayman LL, Woo JG, Villafane J, Celermajer D (2016). Cardiovascular consequences of childhood secondhand tobacco smoke exposure: prevailing evidence, burden, and racial and socioeconomic disparities a scientific statement from the American Heart Association. Circulation..

[8] Israel Ministry of Health (2018). Minister's Report on Smoking in Israel.

[9] Berman T, Barnett-Itzhaki Z, Axelrod R, Keinan-Boker L, Shimony T, Goldsmith R (2018). Socioeconomic inequalities in exposure to environmental tobacco smoke in children in Israel. Environ Int.

[10] Passey ME, Longman J, Robinson J, Wiggers J, Jones LJ (2016). Smoke-free homes: what are the barriers, motivators and enablers? A qualitative systematic review and thematic synthesis. BMJ Open.

[11] Robinson J, Kirkcaldy AJ (2007). ‘You think that I'm smoking and they're not’: why mothers still smoke in the home. Soc Sci Med.

[12] Rosen LJ, Guttman N, Hovell M, Noach M, Winickoff J, Tchernokovski S (2011). Development, design and conceptual issues of project zero exposure. A program to protect young children from tobacco smoke exposure. BMC Public Health.

[13] Rosen LJ, Tillinger E, Rosenblat S, Guttman N, Zucker D, Myers V (2015). Parental receptivity to child biomarker testing for tobacco smoke exposure. Patient Educ Counsel.

[14] Boyatzis RE (1998). Transforming qualitative information: thematic analysis and code.

[15] Braun V, Clarke V (2006). Using thematic analysis in psychology. Qual Res Psychol.

[16] Clarke V, Braun V, Hayfield N, Smith JA (2015). Thematic analysis. Qualitative psychology: a practical guide to research methods.

[17] Charmaz K. Constructing grounded theory: a practical guide through qualitative analysis. London: Sage; 2006.

[18] Rosen L. Historic tobacco legislation in Israel. Isr J Health Policy Res. 2020;9:22. 10.1186/s13584-020-00384-3. 10.1186/s13584-020-00384-3 PMC7199353 32366296

[19] Drehmer JE, Ossip DJ, Nabi-Burza E, Rigotti NA, Hipple B, Woo H (2014). Thirdhand smoke beliefs of parents. Pediatrics..

[20] Van Deusen A, Hyland A, Travers MJ, Wang C, Higbee C, King BA (2009). Secondhand smoke and particulate matter exposure in the home. Nicotine Tob Res.

[21] Spencer N, Blackburn C, Bonas S, Coe C, Dolan A (2005). Parent reported home smoking bans and toddler (18–30 month) smoke exposure: a cross-sectional survey. Arch Dis Child.

[22] Johansson AK, Halling A, Hermansson G, Ludvigsson J (2005). Assessment of smoking behaviors in the home and their influence on children's passive smoking: development of a questionnaire. Ann Epidemiol.

[23] Klepeis N, Ott WR, Switzer P (2007). Real-time measurement of outdoor tobacco smoke particles. J Air Waste Manag Assoc.

[24] Matt GE, Quintana PJE, Hovell MF, Bernert JT, Song S, Novianti N (2004). Households contaminated by environmental tobacco smoke: sources of infant exposures. Tob Control.

[25] Masiero M, Lucchiari C, Pravettoni G (2015). Personal fable: optimistic bias in cigarette smokers. Int J High Risk Behav Addict.

[26] Krosnick JA, Malhotra N, Hyungjung Mo C, Bruera EF, Chang L, Pasek J (2017). Perceptions of health risks of cigarette smoking: a new measure reveals widespread misunderstanding. PLoS One.

[27] Friebely J, Rigotti NA, Chang Y, Hall N, Weiley V, Dempsey J (2013). Parent smoker role conflict and planning to quit smoking: a cross-sectional study. BMC Public Health.

[28] Kovess V, Pilowsky DJ, Boyd A, Pez O, Bitfoi A, Carta M (2013). Parental smoking in the vicinity of children and tobacco control policies in the European region. PLoS One.

[29] Rowa-Dewar N, Lumsdaine C, Amos A (2015). Protecting children from smoke exposure in disadvantaged homes. Nicotine Tob Res.

[30] Fresh. Smokefree Families - How can you keep your home smokefree? . Available from: http://www.smokefreefamilies.co.uk/. Accessed 5 Oct 2019.

[31] EPA. Smoke-free home Community Action Kit. US: Environmental Protection Agency; 2006.

